# Repeat Targeted Prostate Biopsy under Guidance of Multiparametric MRI-Correlated Real-Time Contrast-Enhanced Ultrasound for Patients with Previous Negative Biopsy and Elevated Prostate-Specific Antigen: A Prospective Study

**DOI:** 10.1371/journal.pone.0130671

**Published:** 2015-06-17

**Authors:** Dong Ryul Jang, Dae Chul Jung, Young Taik Oh, Songmi Noh, Kyunghwa Han, Kiwook Kim, Koon-Ho Rha, Young Deuk Choi, Sung Joon Hong

**Affiliations:** 1 Department of Radiology, Severance Hospital, Research Institute of Radiological Science, Yonsei University College of Medicine, Seoul, Republic of Korea; 2 Department of Pathology, Cha Medical College, Gang-Nam Cha Hospital, Seoul, Republic of Korea; 3 Avison Biomedical Research Center; Department of Radiology; Research Institute of Radiological Science, Yonsei University College of Medicine, Seoul, Republic of Korea; 4 Department of Urology, Severance Hospital, Yonsei University College of Medicine, Seoul, Republic of Korea; Queensland University of Technology, AUSTRALIA

## Abstract

**Objectives:**

To prospectively determine whether multi-parametric MRI (mpMRI) - contrast-enhanced ultrasound (CEUS) correlated, imaging-guided target biopsy (TB) method could improve the detection of prostate cancer in re-biopsy setting of patients with prior negative biopsy.

**Methods:**

From 2012 to 2014, a total of 42 Korean men with a negative result from previous systematic biopsy (SB) and elevated prostate-specific antigen underwent 3T mpMRI and real-time CEUS guided TB. Target lesions were determined by fusion of mpMRI and CEUS. Subsequently, 12-core SB was performed by a different radiologist. We compared core-based cancer detection rates (CaDR) using the generalized linear mixed model (GLIMMIX) for each biopsy method.

**Results:**

Core-based CaDR was higher in TB (17.92%, 38 of 212 cores) than in SB (6.15%, 31 of 504 cores) (p < 0.0001; GLIMMIX). In the cancer-positive TB cores, CaDR with suspicious lesions by mpMRI was higher than that by CEUS (86.8% vs. 60.5%, p= 0.02; paired t-test) and concordant rate between mpMRI and CEUS was significantly different with discordant rate (48% vs. 52%, p=0.04; McNemar’s test).

**Conclusion:**

The mpMRI-CEUS correlated TB technique for the repeat prostate biopsy of patients with prior negative biopsy can improve CaDR based on the number of cores taken.

## Introduction

Prostate cancer (PCa) is the second most common cancer and the sixth leading cause of cancer-related death in males worldwide [1, 2]. The 12-core transrectal ultrasound (TRUS)-guided systematic biopsy (SB) is the standard and most common method for the detection of PCa. However, the false negative rate of initial SB may be as high as 47% [3]. A repeat biopsy is frequently required in patients with persisting clinical suspicion of PCa-after negative results from a previous biopsy [4–6]. As the number of re-biopsy rounds increased, the detection rate of PCa gradually decreased [7]. In an effort to improve the cancer detection and reduce false-negative rates, investigators have explored alternative biopsy methods including targeted biopsy (TB) under guidance (correlation) of multi-parametric magnetic resonance imaging (mpMRI) [8]. Recently, mpMRI is widely used for the detection of PCa and has a good detection rate for local cancer lesions within the prostate gland [6, 9–11]. In addition, contrast-enhanced ultrasound (CEUS) images of the prostate gland have shown better ability to detect prostate cancer lesions than standard gray-scale TRUS. Several CEUS studies of the prostate gland have suggested that adding TB to SB could improve cancer detection with a set number of biopsy specimens instead of simply increasing the number of biopsy cores [12, 13].

Although highly sensitive and specific, in-bore MR-guided prostate biopsy is performed selectively due to their procedure length, cost and discomfort to the patient. A potential solution to maximize the sensitivity is to acquire pre-biopsy diagnostic mpMRI and to correlate them to the real-time CEUS images acquired during the TRUS-guided TB procedure. With this protocol, the diagnostic power of prostate mpMRI could be fully coupled to the flexible and rapid US-guided procedure.

The objective of our study is to evaluate prospectively the pre-acquired mpMRI-correlated, real-time CEUS guided TB technique for the repeat prostate biopsy of patients with elevated PSA levels. We hypothesized that the TB method will double the core-based detection rate of the conventional SB method..

## Materials and Methods

### Patients

**Fig 1:**
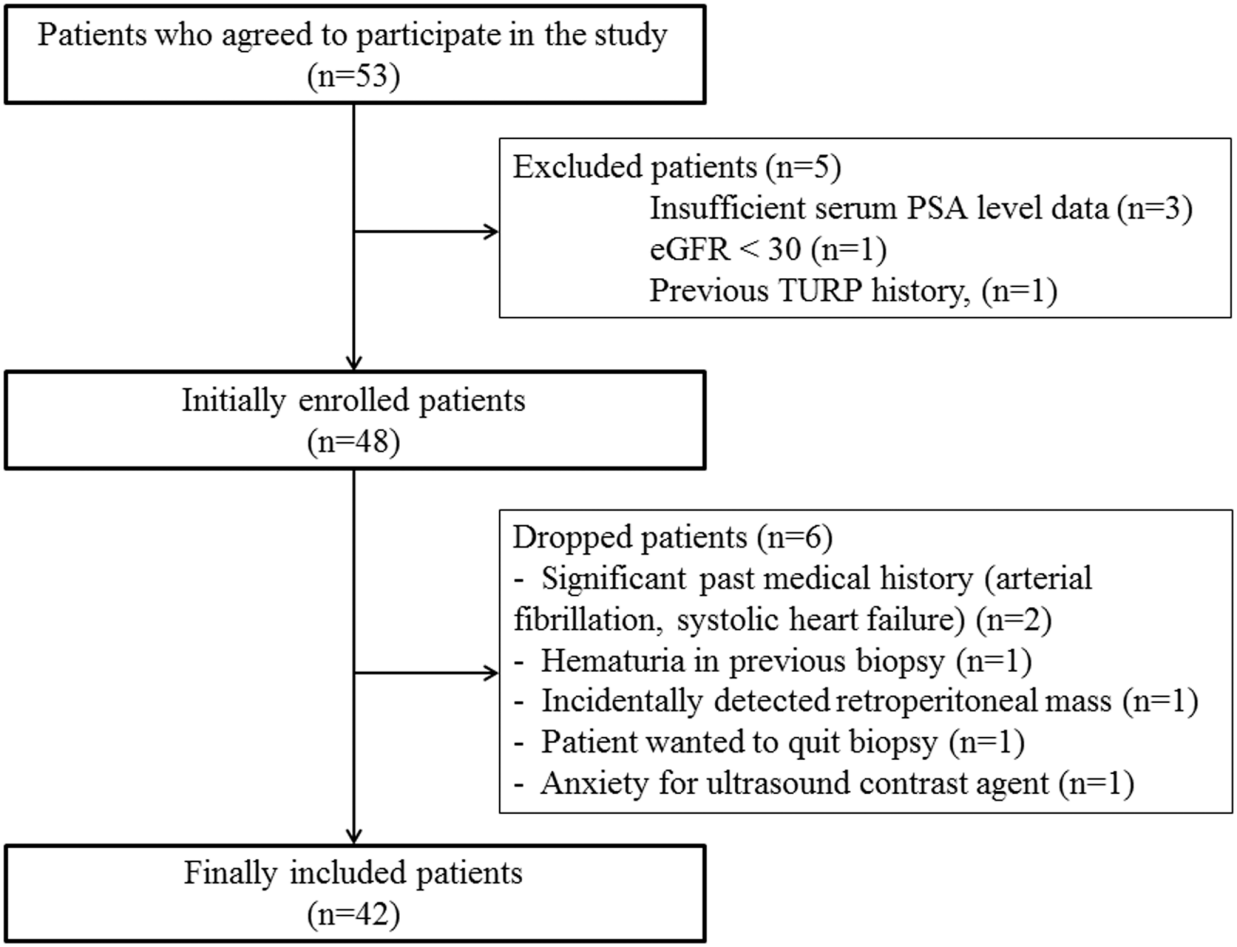
Flow chart of the patient enrollment.

Patients who tested negative in a previous conventional 12-core TRUS-guided prostate biopsy and were scheduled for repeated biopsies due to persistent suspicion of PCa based on a continuously elevated serum PSA level (more than 4 ng/ml) were eligible for this study. Study enrollment started in October 2012 and finished July 2014. Fifty-three patients agreed to participate, but 5 were excluded for reasons stated in Fig 1. Thus 48 patients were enrolled, with 6 additional patients dropping out of the study (Fig 1.). The exclusion criteria were patients previously diagnosed with PCa, with a history of transurethral resection of prostate (TURP), prostatectomy or radiation therapy to the pelvis for any other malignancy, or contraindication to mpMRI or CEUS (pacemaker, magnetic implants, gadolinium-based MRI contrast allergy, impaired renal function with glomerular filtration rate < 30 ml/min). Finally, 42 patients were included prospectively, and this number was estimated according to the generalized linear mixed model (GLIMMIX) method for the statistical prediction of a meaningful number of patients via statistical power analysis [14]. The detailed statistical modeling was summarized in the S1 Table. Yonsei University Health System, Severance Hospital, Institutional Review Board approved the entire study. Each patient signed the informed consent.

### Multi-parametric MR imaging

To identify suspected PCa lesions, patients underwent mpMRI using a 3T MR scanner (Siemens, Trio Tim, Erlangen, Germany) without endorectal coil after injection of MR contrast media, Gadoterate meglumine (Dotarem, Guerbet, Roissy CdG, France) (Table 1). MR imaging was performed at least six months after any previous biopsy, to avoid the post-biopsy hemorrhage of the prostate. Images were reviewed by a specialized radiologist with ten years of experience in prostate mpMRI using the validated Prostate Imaging Reporting and Data System (PI-RADS) [15–17]. After reviewing the mpMRI, the radiologist chose candidates among equivocal (PI-RADS 3) or intermediate/high-risk (PI-RADS 4/5) lesions on mpMRI and ranked them depending on the scores. If any cases had multiple suspicious lesions on review, we limited these to less than 3.

**Table 1:** MR protocol of multiparametric MRI of the prostate who underwent contrast-enhanced US guided biopsy.

1.	T2WI axial, coronal, sagittal: TR, 3800 msec; TE, 98 msec; slice thickness, 4 mm; 448 x 314 matrix; FOV, 150x150 mm.
2.	T1WI axial, before contrast material injection: TR, 700 msec; TE, 11 msec; slice thickness, 5 mm; 320 x 288 matrix; FOV, 160x160 mm.
3.	DWI axial with a single-shot-echo-planar imaging: b-values of 0, and 1000 s/mm2; TR, 4400 msec; TE, 86 msec; slice thickness; 4 mm; 192 x 192 matrix; FOV, 240x240 mm.. ADC maps were automatically calculated by the scanner software.
4.	DCE T1WI axial during an intravenous injection of gadolinium-based contrast material at 0.1 mmol/kg: TR, 3.44 msec; TE, 1.19 msec; thickness, 2 mm; matrix, 320×192; FOV, 360x252 mm. The Ktrans map for each voxel was finally overlapped over T2-weighted images*.

T1WI and T2WI = T1- and T2-weighted images, TR = repetition time, TE = echo time, msec = millisecond, FOV = field of view, DWI = diffusion weighted images, ADC = apparent diffusion coefficient, DCE = dynamic contrast enhanced, Ktrans = transfer constant.

* The fitting of concentration versus time curves was performed based on theoretical models by Tofts. Perfusion-related parameters including Ktrans were derived by the curves [18]. We used commercial software (Tissue4D; Siemens healthcare, Erlangen, Germany) in the construction of perfusion map images.

### Contrast-enhanced US (CEUS) imaging and biopsy

Within one week after imaging by mpMRI, transrectal CEUS was performed on each patient. The contrast agent SonoVue (Bracco, Milan, Italy) was used in conjunction with a Siemens S2000 US system (Siemens Medical Solution, Mountain View, CA, USA) in a contrast-specific imaging mode called Cadence contrast pulse sequencing (CPS) technology (Siemens Medical Solution, Mountain View, CA, USA) (S2 Table). If there was a suspected lesion on first bolus of SonoVue, the CEUS-based suspicious lesions were compared with the lesions already selected on mpMRI by the radiologist who reviewed the mpMRI and performed transrectal CEUS simultaneously [19]. The target sites were decided by taking into account both mpMRI and CEUS images. And then a repeated bolus was used for exact targeting. We targeted suspicious lesions subjectively during first round real-time CEUS and confirmed that lesion in a second CEUS after 3 to 5 minutes from the 1st injection.

**Fig 2:**
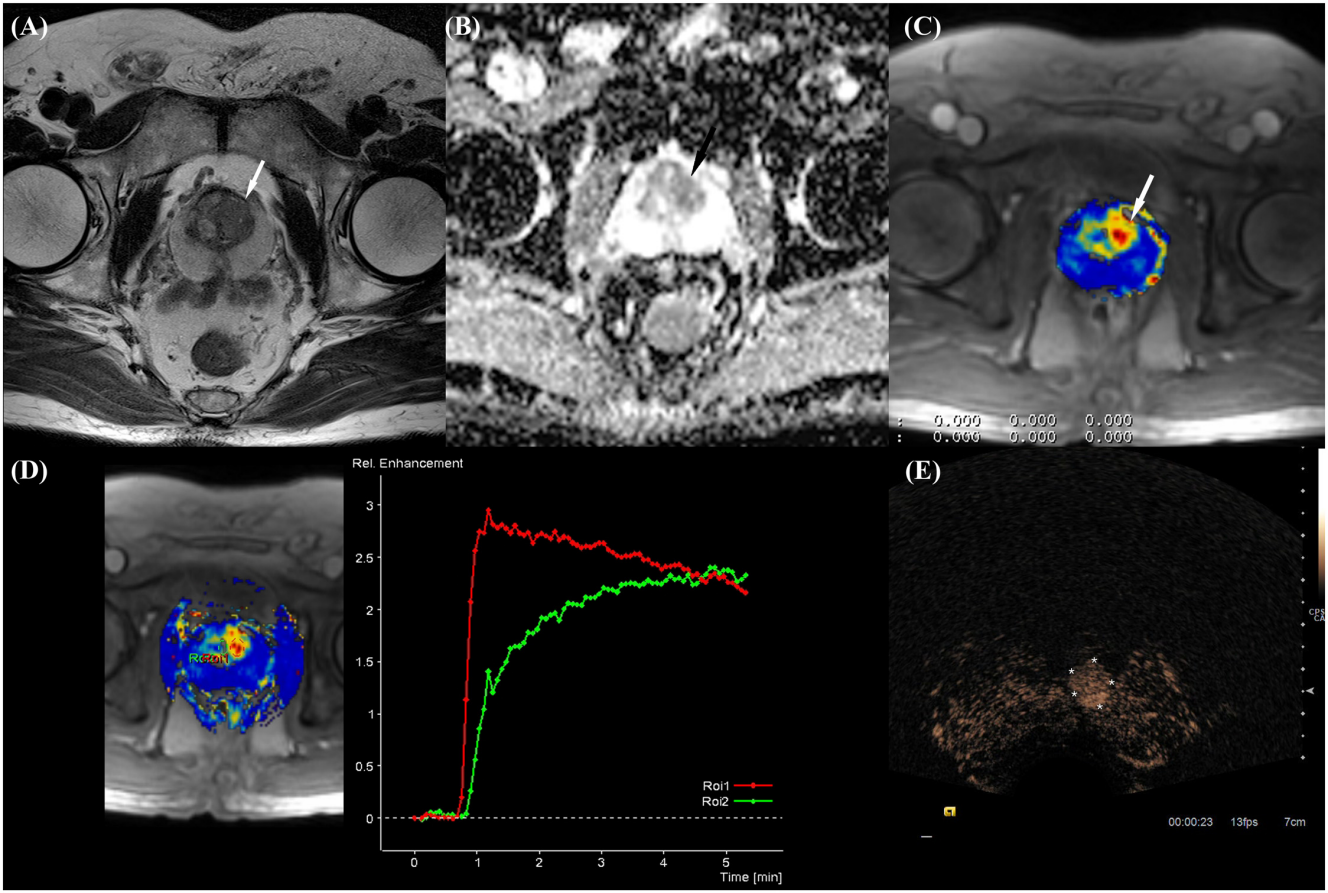
Prostate cancer in a 73-year-old man with a PSA level of 12.3 ng/mL and a history of a negative biopsy findings. A. Axial T2-weighted image in a 71-year-old man with prostate cancer in the left TZ shows homogeneous low signal intensity, ill-defined margins, and lack of a hypointense capsule (arrow). B. Corresponding ADC map shows restricted diffusion as area of low signal intensity (arrow). C. Axial perfusion map shows focal enhancement in left TZ (arrow). D. Time intensity curve shows red, type 3 enhancement curve (red circle: cancer focus). Green curve is type 1 enhancement pattern (green circle: normal tissue). E. Axial transrectal US image acquired after contrast material injection shows the corresponding an asymmetric early wash-in area on contrast pulse sequence–mode images (outlined by asterisks). Biopsy was performed targeting this area, and we were able to confirm the presence of cancer. Histopathologic examination indicated a prostate carcinoma with a Gleason score of 6 (3+3) at targeted biopsy (TB) only.

TB for PCa was performed as follows: first, correlated lesions on both mpMRI and CEUS were targeted (Fig 2.); second, CEUS-only positive lesions were targeted; finally, mpMRI-only suspicious lesions were taken by TB under TRUS guidance. If multiple lesions were detected, we also performed TB on each lesion a total of not more than six times. If there was no suspicious lesion on both images, an additional six randomized biopsies were performed in both transitional zones and peripheral zones. After TB, conventional TRUS-guided 12-core SB was performed using an extended sextant biopsy scheme by a different radiologist (YTO) who was unaware of the mpMRI/CEUS findings [20]. All biopsies were performed using an 18-gauge spring-loaded Acecut biopsy gun (TSK Laboratories, Nagoya, Japan). All biopsy cores were coded with number and group (TB or SB) and sent for pathologic examination. Biopsy cores were reviewed by one of the cytopathologists specializing in the genitourinary system and reported either as cancer with an assigned Gleason score or as benign prostatic tissue.

### Statistical analysis

The prostate cancer detection rate (CaDR) based on core numbers was calculated in addition to the overall patient number-based CaDR. We compared the core-based detection rate of each biopsy method (TB versus SB) using GLIMMIX with correlated binary outcomes in this clustered data. To determine whether differences in the Gleason score between SB and TB were significant, the Wilcoxon’s signed-rank test was performed. In the analysis of cancer-positive TB cores, we performed McNemar’s test between mpMRI and CEUS findings in each cores. All reported P values are one-sided, and a significant level of 5% was used. All analyses were conducted using MedCalc software for Windows (MedCalc Software version 12.7.5, Mariakerke, Belgium) and SPSS 20.0 (released 2011, IBM statistics for Windows version 20, IBM Corp., Armonk, NY) for GLIMMIX analysis.

## Results

Table 2 lists summary statistics relating to the patients. Overall, PCa was detected in 18 of 42 patients (42.9%). Fourteen of 18 patients (77.8%) with PCa had clinically significant cancer (Gleason >6 or Gleason 6 with >50% PCa per core or >2 cores PCa) [21]. Fifteen patients with PCa (83.4%) had predominant Gleason 3 architecture (Gleason 3 + 3 or 3 + 4). The results of the patient-based CaDR are summarized in Table 3. The mean number of all biopsy cores per participant was 17.8 (Range: 15–18).

**Table 2:** Patient demographics of the real-time contrast enhanced ultrasound guided biopsy.

	All Subjects(n = 42)*	PCa proven(n = 18)*
Age, year (mean±SD)	62.7±10.0	65.8±6.69
Median (range)	65(28 ~ 77)	68(54 ~76)
PSA(ng/mL)		
Median (range)	9.77 (4.3 ~ 99.1)	8.76 (4.3 ~ 99.1)
4–9.9	22 (52.4%)	11(61.1%)
More than 10	20 (47.6%)	7 (38.9%)
Prostate volume (mL)	44.01±20.99	33.96±11.77
Median(range)	39.5(12.5 ~ 88.7)	32.6(12.5 ~ 51.0)
< 30 cc	13 (31.0%)	8 (44.4%)
> = 30 cc	29 (69.0%)	10 (55.6%)
DRE		
Normal	15 (35.7%)	7 (38.9%)
Abnormal	27 (40.5%)	11 (61.1%)
mpMRI score		
PI-RADS 1/2	9	0
PI-RADS 3	14	5
PI-RADS 4/5	19	13
CEUS findings		
CEUS negative	10	3
CEUS positive	32	15

PCa = Prostate cancer; PSA = Prostate specific antigen; DRE = digital rectal examination; mpMRI = multiparametric Magnetic resonance imaging; PI-RADS = Prostate Imaging Reporting and Data System; CEUS = contrast enhanced ultrasound;

* No. of patients

**Table 3:** Patient based comparison of cancer detection rate between systematic biopsy group and targeted biopsy group.

	Target biopsy		
Systematic biopsy	Negative	Positive	Total (%)
Negative	24	5	29 (69.0)
Positive	5	8	13 (31.0)
Total (%)	29 (69.0)	13 (31.0)	42

CaDR based on biopsy cores are summarized in Table 4. A total of 212 TB cores and 504 SB cores was obtained from 42 patients. CaDR was significantly (p < 0.0001; GLIMMIX) higher in the TB cores (17.92%, 38 of 212 cores) than in the SB cores (6.15%, 31 of 504 cores). The Gleason score detected by the two techniques was not significantly different (P = 0.84; Wilcoxon signed-rank test). In the analysis of cancer-positive TB cores (Table 5), CaDR with suspicious lesions by mpMRI (PI-RADS 3/4/5) was much higher (86.8%, 33 of 38 cores) than that by CEUS (60.5%, 23 of 38 cores). Five discordant cores, however, revealed CEUS positive and mpMRI negative finding. And, concordant rate between mpMRI and CEUS was not significantly different with discordant rate (48% vs. 52%, p>0.01).

**Table 4:** Core based comparison of cancer detection between systematic biopsy group and targeted biopsy group.

	TB (%)	SB (%)	P (95 C.I)
ALL	17.92 (38/212)*	6.15 (31/504)*	< 0.0001(4.554~15.105)
PIRADS 3/4/5	28.44 (33/116)*	n/a	
PIRADS 1/2	5.21 (5/96)*	n/a	
CEUS positive	18.11 (23/127)*	n/a	
CEUS negative	17.64 (15/85)*	n/a	
Gleason sum(n, %)			
Benign	174 (82.1)	473 (93.8)	
GS 6	23 (10.9)	27(5.4)	
GS 7	7 (3.3)	2(0.4)	
GS 8–10	8 (3.7)	2(0.4)	

TB = Target biopsy; SB = Systematic biopsy; PI-RADS = Prostate Imaging Reporting and Data System; n/a = not applicable; CEUS = contrast enhanced ultrasound

* Positive/Total cores (cancer detection rates)

**Table 5:** Agreement between mpMRI and CEUS in positive TB cores (Numbers of cores containing cancer detected by targeted biopsy).

	mpMRI		
CEUS	Negative(PIRADS 1/2)	Positive(PIRADS 3/4/5)	Total (%)
Negative	0	15	15 (39.5)
Positive	5	18	23 (60.5)
Total (%)	5 (13.2)	33 (86.8)	38

CEUS = contrast enhanced ultrasound; mpMRI = multi-parametric MRI; TB = Target biopsy; PI-RADS = Prostate Imaging Reporting and Data System

## Discussion

We performed cognitive fusion between mpMRI and CEUS, which was simple, quick and required no additional equipment beyond the MRI and a CEUS facility. This method may overcome the disadvantage of simple cognitive fusion using conventional T2WI and ultrasound because perfusion mapping derived from DCE-MRI and real time CEUS images offers additional similar perfusion information in prostate tissues and is helpful in the detection and real-time registration of focal hypervascular lesions so that lesions can be targeted more precisely. The increase in microvessels in prostate cancer enables CEUS to improve vascular imaging and resolution [22]. By adding CEUS for TB, diagnostic performance, especially sensitivity, increases. Tumors located at the peripheral zone, with higher Gleason score and larger size, are more likely to become markedly enhanced [23]. Although CEUS improves PCa detection in rebiopsy setting, the sensitivity of CEUS-only biopsy is reduced in small, low-grade tumors, centrally located lesions and large infiltrating prostate tumors [24]. In addition, CEUS-combined TRUS guided targeted biopsy in previous years has not considered clinically useful [25]. After TB for MR-CEUS correlated lesions, additional TB cores were taken from discrepant lesions, mpMRI positive and CEUS negative lesions, or vice versa. Hence, this method could cover lesions from standard cognitive fusion biopsy (MRI-TRUS) as well as lesions hidden from CEUS or mpMRI. In addition, we could have another chance at detecting cancerous tissue which appear a ‘normal’ on mpMRI; however, this could still be problematic, as false-negative aspects of prostate MRI are not yet known. Among discrepant lesions between mpMRI and CEUS, mpMRI-only positive cores showed markedly higher detection rate than CEUS-only positive cores. This result indicates that we can increase the prostate CaDR by targeting suspicious lesions with a bias toward pre-biopsy mpMRI over CEUS. In addition, we found that the 15 mpMRI-only positive cores were mainly located in anterior portion of transitional zone and anterior fibromuscular stroma (60%, 9/15), which were consistent with those of a previous study by Pepe et al [26]. Our mpMRI-CEUS-correlated TB showed an improved CaDR with fewer cores than SB; thus, this method may be comparable to saturation biopsy techniques with a higher number of samples (20 cores; CaDR, 25%–41%) [27–29]. Also, MR-CEUS guided biopsy method with cognitive correlation was significantly more cost-effective than the inbore-MR guided biopsy, since the patient pays extra for the contrast agent instead of the inbore-MR biopsy equipment.

There was a technical limitation of CEUS. Because of a limited temporal window of CEUS for scanning entire prostate, biopsy had to be performed in very short time duration. As the prostate was larger, we had less of a chance to observe the enhancing pattern of the entire prostate. Follow up TB or SB will be planned in patients who were negative in this study. Since we carried out an extended biopsy (12 cores) in the control group instead of saturation biopsy, we could not perform a comparison between TB and saturation biopsy. Two of 5 patients who have missed PCa on TB cores were clinically significant.

We initially hypothesized for detection rate to be twice by using this multi-modality-correlated, imaging-guided TB technique. We achieved the improvement in CaDR based on the number of cores taken. In conclusion, the mpMRI-CEUS-correlated imaging-guided TB technique for the repeat prostate biopsy of patients with suspected PCa can be used to improve diagnostic accuracy.

## Supporting Information

S1 File Full dataset. (XLSX)

S1 Table The parameters of generalized linear mixed model (GLIMMIX) for the statistical prediction of a number of patients via statistical power analysis [14]. (DOCX)

S2 Table Contrast-Enhanced US (CEUS) protocol using Cadence contrast pulse sequencing (CPS) technology (Siemens Medical Solution, Mountain View, CA, USA). (DOCX)
