# Supplementary material for: Repeat Targeted Prostate Biopsy under Guidance of Multiparametric MRI-Correlated Real-Time Contrast-Enhanced Ultrasound for Patients with Previous Negative Biopsy and Elevated Prostate-Specific Antigen: A Prospective Study
Source: PLoS One. 2015 Jun 17;10(6):e0130671. doi: 10.1371/journal.pone.0130671 (PMC4471162; doi:10.1371/journal.pone.0130671)
Supplement: S1 Table — (DOCX) [file pone.0130671.s002.docx]

**Supporting Information**

**S1 Table. The parameters of generalized linear mixed model (GLIMMIX) for the statistical prediction of a number of patients via statistical power analysis [14].**

| 1. | Biopsy core-based detection rate of our institution was determined as 0.06, which derived from statistics in our urology clinics from 2009 to 2011. Our goal of detection rate would be double of that resulted in 0.12. |
| --- | --- |
| 2. | Estimates are for the minimum total sample required in two groups, with a sample allocation ratio of 1:1, for comparison of event probabilities. |
| 3. | We estimate the variance of the random effect to be 1 and the within-subject correlation rho to be 0.03210 for the total sample. So, estimates are for setting the random effects to G= 1, type I error to 0.05, and type II error to 0.20, which provides model-correlated outcomes with different types of distributions. (80 % power at 5% level of significance), which provides model-correlated outcomes with different types of distributions. The sample size finally estimated was 42. |
