# Supplementary material for: Repeat Targeted Prostate Biopsy under Guidance of Multiparametric MRI-Correlated Real-Time Contrast-Enhanced Ultrasound for Patients with Previous Negative Biopsy and Elevated Prostate-Specific Antigen: A Prospective Study
Source: PLoS One. 2015 Jun 17;10(6):e0130671. doi: 10.1371/journal.pone.0130671 (PMC4471162; doi:10.1371/journal.pone.0130671)
Supplement: S2 Table — (DOCX) [file pone.0130671.s003.docx]

**Supporting Information**

**S2 Table. Contrast-Enhanced US (CEUS) protocol using Cadence™ contrast pulse sequencing (CPS) technology (Siemens Medical Solution, Mountain View, CA, USA).**

| 1. | The CPS mechanical index (MI) was determined to be between 0.14-0.21. |
| --- | --- |
| 2. | An intravenous line was inserted in the antecubital vein with a 20-22G needle to inject the contrast agent. |
| 3. | Contrast agent powder was mixed with 5 mL of 0.9% normal saline, and 2.4 mL was injected intravenously with 5-10 mL saline flushing. |
| 4. | Criteria to evaluate the suspected areas in CEUS images [19]  1) Rapid or increased contrast enhancement compared to surrounding parenchyma  2) Asymmetric appearance of intraprostatic vessels compared to contralateral lobe of prostate on the same image  3) A perfusion defect in the peripheral zone was also considered suspicious lesions. |
